# Intrahost Norovirus Evolution in Chronic Infection Over 5 Years of Shedding in a Kidney Transplant Recipient

**DOI:** 10.3389/fmicb.2018.00371

**Published:** 2018-03-02

**Authors:** Andrej Steyer, Tilen Konte, Martin Sagadin, Marko Kolenc, Andrej Škoberne, Julija Germ, Tadeja Dovč-Drnovšek, Miha Arnol, Mateja Poljšak-Prijatelj

**Affiliations:** ^1^Faculty of Medicine, Institute of Microbiology and Immunology, University of Ljubljana, Ljubljana, Slovenia; ^2^Faculty of Medicine, Institute of Biochemistry, University of Ljubljana, Ljubljana, Slovenia; ^3^Department of Nephrology, University Medical Centre Ljubljana, Ljubljana, Slovenia; ^4^Blood Transfusion Centre of Slovenia, Ljubljana, Slovenia

**Keywords:** norovirus, molecular evolution, chronic infection, solid organ transplantation, HBGA binding

## Abstract

Noroviruses are the leading cause of acute gastroenteritis, and they can affect humans of all age groups. In immunocompromised patients, norovirus infections can develop into chronic diarrhea or show prolonged asymptomatic virus shedding. Chronic norovirus infections are frequently reported for solid organ transplant recipients, with rapid intrahost norovirus evolution seen. In this report, we describe a case of chronic norovirus infection in an immunocompromised patient who was followed up for over 5 years. The purpose of the study was to specify the norovirus evolution in a chronically infected immunocompromised host and identify possible selection sites in norovirus capsid protein. During the follow-up period, 25 sequential stool samples were collected and nine of them were selected to generate amplicons covering viral RNA-dependent RNA polymerase (RdRp) and viral capsid protein (VP1) genes. Amplicons were sequenced using next-generation sequencing. Single nucleotide polymorphisms were defined, which demonstrated a nearly 3-fold greater mutation rate in the VP1 genome region compared to the RdRp genome region (7.9 vs. 2.8 variable sites/100 nucleotides, respectively). This indicates that mutations in the virus genome were not accumulated randomly, but are rather the result of mutant selection during the infection cycle. Using ShoRAH software we were able to reconstruct haplotypes occurring in each of the nine selected samples. The deduced amino-acid haplotype sequences were aligned and the positions were analyzed for selective pressure using the Datamonkey program. Only 12 out of 25 positive selection sites were within the commonly described epitopes A, B, C, and D of the VP1 protein. New positive selection sites were determined that have not been described before and might reflect adaptation of the norovirus toward optimal histo-blood-group antigen binding, or modification of the norovirus antigenic properties. These data provide new insights into norovirus evolutionary dynamics and indicate new putative epitope “hot-spots” of modified and optimized norovirus–host interactions.

## Introduction

Noroviruses (NVs) are the leading cause of sporadic and epidemic acute gastroenteritis in humans, with the young and the elderly being the most vulnerable populations. Annually, there are an estimated 677 million cases of NV diarrhea worldwide, which results in >200,000 deaths globally (Pires et al., 2015). NVs are small, non-enveloped, icosahedral viruses with a genome of 7,500 nucleotides of positive polar single-stranded RNA. The NV genome encodes several non-structural proteins within open-reading frame (ORF)1, the major structural viral capsid protein (VP)1 within ORF2, and minor capsid proteins within ORF3 (Thorne and Goodfellow, 2014).

NVs are known to interact with human histo-blood-group antigens (HBGAs) and to show high specificity for certain types of these antigens (Rockx et al., 2005b; Schroten et al., 2016). Also, NV pathogenesis is dependent on the host secretor (α1,2-l-fucosyltransferase) status, and the ABO and Lewis (LE) blood groups. Shanker et al. (2011) showed that temporal sequence variations do not affect the binding of monofucosyl ABH HBGAs, but that they can modulate the binding strength of difucosyl Lewis (Le) HBGAs, and can thus contribute to epochal evolution through the potentiated targeting of new variants to Le-positive, secretor-positive individuals (Shanker et al., 2011).

According to the Le phenotype and the secretor gene (*FUT2*), individuals can be classified as secretors and non-secretors (GRUBB, 1948). Le(a^+^b^−^) red cells come from ABH non-secretors, Le(a^−^b^+^) red cells from ABH secretors, and Le(a^−^b^−^) red cells from ABH secretors and non-secretors (Daniels, 2002). Thus, the two alleles of *FUT2, Se* (dominant) and *se* determine the presence or absence of ABH substances in body fluids (Holbro et al., 2015). The ABH-secretor status can be determined serologically by Le^b^ antigen positivity, or by molecular genetic analysis of the *FUT2* gene.

The NV capsid is formed from ninety VP1 dimers, where each VP1 monomer includes a shell (S) domain and a protruding (P) domain. The P domain is further divided into the P1 (residues 226-278, 406-520) and P2 (residues 279-405) subdomains (Prasad et al., 1999). As the P domain is involved in antibody recognition (Lindesmith et al., 2011), its rapid evolution is probably driven by the host immunity selection pressure that generates new epidemic strains with modified blockade epitopes and altered HBGA binding properties (Lochridge and Hardy, 2007; de Rougemont et al., 2011; Tan and Jiang, 2011; Schroten et al., 2016). The P domain is the region of the capsid that accumulates the most mutations also in immunocompromised patients (Bull et al., 2012; Debbink et al., 2014). Residues of the P domain that are repeatedly identified as evolving sites in different NV GII.4 strains (i.e., genogroup II, genotype four; see below) belong to epitope A (residues 294, 296-298, 368, 372), epitope D (residues 393-395), and epitope E (residues 407, 412, 413) (Allen et al., 2009; Debbink et al., 2012; Lindesmith et al., 2012b). Recently, Debbink et al. (2014) expanded epitope A to seven residues by including residue 373, and Lindesmith et al. (2012a) proposed new sites as potential for epitopes B (residues 333, 382) and C (residues 340, 376).

The parts of the P-domain surface that are more conserved are the two HBGA binding pockets. These pockets can interact with different types of HBGAs, and thus mediate NV infection (Rockx et al., 2005a). Each of the two HBGA binding pockets spreads across both VP1 monomers, and they include sites I and II. Residues 343-345, 347, and 374 of one VP1 monomer represent part of site I, and residues 390′-393′, and 442′-444′ of the other VP1' monomer represent part of site II. These residues bind HBGAs with direct or water-mediated interactions (Shanker et al., 2011; Singh et al., 2015).

NVs are classified into at least six genogroups, and a tentative seventh genogroup, according to this VP1 capsid protein (Vinjé, 2015). They are further subdivided into genotypes that share ≥80% amino-acid identity compared with the complete capsid gene sequence (Koopmans et al., 2000). The most common NVs in humans are from genogroup II, and specifically of genotype four (i.e., GII.4), which is the genotype that has shown numerous epidemic variants over the last 20 years (White, 2014). These appear in populations with periodic epidemic peaks, as every 2–4 years. New epidemic or pandemic GII.4 variants appear and generally spread very rapidly through populations and across continents. It appears that low cross-protection by specific anti-NV humoral immune responses have the major roles in NV epidemics and pandemics (Debbink et al., 2013).

Special attention needs to be paid to immunocompromised persons, where NVs can cause prolonged infections, with NV shedding in stools (Green, 2014; Woodward et al., 2017). Chronic NV infections have already been reported in various studies, with these mainly seen in solid organ transplant recipients. In these cases, the patients report diarrhea at the acute stages of infection, which is followed by disappearance of the clinical symptoms, or alternatively they report prolonged periods of diarrhea (Echenique et al., 2016). The clinical presentation of such chronic infection is often correlated with modifications to their immunosuppressive therapy.

The important parameter in chronic NV infection is the viral evolution within the host. Chronic infections can contribute new data in studies into the changing patterns of NV molecular characteristics, particularly in terms of the VP1 capsid protein. It was previously suggested that chronically infected patients can produce NV variants that can have pandemic potential (Karst and Baric, 2015). The evolutionary rate in these patients is high, and it remains unknown whether such infections represent positive selection of variants by specific immune systems, or only the generation of NV variants that can produce infective NV particles because the immune system is suppressed, and thus does not influence the selection of the escape mutants.

Here, we present a case of chronic NV infection in a patient shortly after kidney transplantation. This patient was followed up for over 5 years for NV variant analysis at the level of the nucleotide and deduced amino-acid sequences. With this study, we analyzed the possible immune-driven NV evolution in a chronically infected immunocompromised host, and explored whether strong positive selection of NV mutants is present in such cases.

## Materials and methods

### Case description

This case is a 48-year-old woman who had end-stage renal disease secondary to IgA nephropathy, a history of arterial hypertension, secondary hyperparathyroidism, and renal anemia, and who had required hemodialysis since June 2008. She received a kidney transplant from a deceased donor in June 2009, with induction of immunosuppression with basiliximab. Cyclosporine, methylprednisolone, and mycophenolate mofetil provided her maintenance immunosuppression therapy. The post-operative period was uneventful, and the patient was discharged 10 days after transplantation.

Two days after discharge, the patient presented with acute diarrhea, with the passing of watery stools two to three times per day. She showed no fever, vomiting, or stomach pain, and no blood or mucus was noted in her stools. Her white cell count was 22.8 × 10^9^/L and her neutrophil count was 19.2 × 10^9^/L; her C-reactive peptide remained in the normal range. Examination of her stools for enteric viruses using real-time RT-PCR was positive for NV GII. Stool culture and *Clostridium difficile* tests were negative. The diarrhea resolved spontaneously in a few days. The patient did not recall having bowel problems prior to her kidney transplantation.

During regular follow-ups, the patient reported occasional intermittent periods of loose stools, up to three times daily. In August 2009, the treatment with cyclosporine was replaced with tacrolimus, due to hypertrichosis. The patient stools were further examined 25 times between June 2009 and April 2014, to monitor for the presence and/or shedding of viruses, and to prevent nosocomial transmission. For all of the 25 samples examined using molecular tests, NVs were detected. Her kidney graft function was excellent during this period, although persistent lymphopenia was noted in her laboratory tests. Eight years post-transplantation, the patient still reports occasional loose stools up to four times daily, but with otherwise satisfactory health conditions. Her kidney graft function remains excellent (serum creatinine, 65 μmol/L; estimated glomerular filtration rate (CKD-EPI), 90.6 mL/min/1.73 m^2^), with no urine abnormalities. The patient remains on triple immunosuppression therapy with tacrolimus, methylprednisolone, and mycophenolate mofetil.

The stool samples collected from the patient during the regular follow-ups at the transplant clinic were sent immediately to the Institute for Microbiology and Immunology, Faculty of Medicine, University of Ljubljana (Slovenia) for diagnostics for enteric pathogens. Blood samples for determination of her LE blood group and secretor status were also taken, with written consent of the patient. The study was approved by the Slovenian National Medical Ethics Committee (N° 0120-545/2016-2).

### NV genotyping

The NV genotypes were determined from all of the nine samples from the patient that went through this genotyping analysis. All of the stool samples were processed equally to prepare 10% (w/v) stool suspensions in phosphate-buffered saline, which was used in the iPrep Viral DNA/RNA extraction protocol (Thermo Fischer Scientific, Waltham, MA, USA). Real-time RT-PCR (Kageyama et al., 2003) was used to detect NV RNA. The extracted nucleic acids were used for RT-PCR amplifications of a short fragment of the RNA-dependent RNA polymerase (RdRp) and VP1 genes, as described previously (Kojima et al., 2002; Vennema et al., 2002). Short genome fragment sequences were obtained for genotype determination, using Sanger sequencing and the NV genotyping tool at http://www.rivm.nl/mpf/typingtool/norovirus/ (June, 2017, Kroneman et al., 2011).

### Quantitative RT-PCR for GII.4 NV

The real-time RT-PCR used for the detection of the NV genome was further used for the quantification system (RT-qPCR). The RNA standard was prepared from a 234 bp segment that contained the real-time amplification region. For the preparation of the RNA standard, primer pairs NV2-F (5′-GGH CCA KCA TTY TAC AGC AA-3′) and NV2-R (5′-TTR TTG AYC TCT GGV ACG AG-3′) were constructed for amplification of the 4922–5155 bp region, according to the deposited NV genome of the Camberwell strain (GenBank accession number, AF145896). The DNA amplicon for standard preparation was cloned into the pJET1.2/blunt plasmid vector with the T7 promoter (Thermo Fischer Scientific, Waltham, MA, USA). The RNA transcript was obtained after plasmid purification and XbaI digestion (Thermo Fischer Scientific), using TranscriptAid T7 High Yield Transcription kits (Thermo Fischer Scientific). The RNA transcript was purified through LiCl precipitation, and the quantity of the transcript was determined using a NanoDrop spectrophotometer (Thermo Fischer Scientific), to obtain the copy-number concentration of the transcript.

### Generation of amplicons and next-generation sequencing

The NV genome amplicon for virus population studies in chronic infected hosts was obtained with primer-pair construction that allowed amplification of the total RdRp and VP1 genes. The primer pairs were constructed as follows: NV-GII.4-RdRp-F 5′- TGY CCC TAY ATC TAC AAG AG-3′ and NV-GII.4-VP1-R 5′- TCA ATT TGT GCT TGG AGC AT-3′, which corresponded to positions 3449–6912 according to the genome of the NV Hu/GII-4/Saga1/2006/JP strain (GenBank accession number, AB447456). This resulted in an amplified genome segment of 3,465 nucleotides. The genome fragment was amplified using RT-PCR and SuperScript III RT/Platinum Taq High Fidelity Enzyme Mix (Thermo Fischer Scientific), according to the standard protocol. The PCR products were purified using Wizard SV Gels and PCR Clean-up purification kits (Promega, Madison, WI, USA), and they were stored at −20°C until the library preparation and next-generation sequencing (NGS).

The library of the amplicons was prepared using Nextera XT DNA Library Preparation kits (Illumina, San Diego, CA, USA), following the manufacturer instructions, which generated 300 bp fragments. The sequencing was performed on MiSeq (Illumina), generating paired-end reads. The reference strain was constructed as the consensus of *de-novo* assembly of NGS reads from the first sample (p1t1) obtained from the patient, and was used as the reference (index) sequence for the patient throughout the remaining samples (p1t2-p1t9).

### Single nucleotide polymorphism analysis, haplotype construction, and phylogenetic analysis

The reads of the individual sequential samples were mapped to the consensus sequence of the first sample (p1t1) for the single nucleotide polymorphism (SNP) analysis. Read mapping and SNP analysis were carried out using the Geneious software (Biomatters Ltd, Auckland, New Zealand), with the following criteria: coverage of >100-fold; nucleotide variant presented in >5%; and read quality of Q30 ≥ 85.7%. The SNP accumulation was compared across the RdRp and VP1 gene regions.

For the VP1 gene region, the haplotypes were constructed for each of the samples included in the NGS analysis, and used for subsequent analysis of VP1 variability at the amino-acid level and for neutralization epitope modifications throughout the patient infection period. The Shorah software was used to reconstruct the VP1 haplotypes, spanning a region of 1,618 bp. The window size of 141 was set, and the default number of window shifts was used (step size, 47). For further analysis, only the haplotypes with calculated frequency of ≥0.05 were included. The haplotype frequencies for each sample were summed and normalized to 1.0.

The haplotype nucleotide sequences of the VP1 gene were aligned and a phylogenetic tree was constructed using the maximum likelihood method, based on the Kimura 2-parameter model. Phylogenetic analysis was conducted using Mega, version 6 (Tamura et al., 2013).

All haplotype sequences were deposited in GenBank under accession numbers MG546825-MG546862.

### Determination of patient LE blood group and secretor status

#### Serological determination of LE blood group

Serologic typing for the determination of the patient LE blood group, and consequently the possible determination of the patient secretor status, was performed using the gel test ID system with monoclonal antibodies for the Le^a^ and Le^b^ antigens, according to the manufacturer protocol (Bio-Rad, Switzerland).

#### Molecular determination of secretor status

The patient secretor status was determined through molecular analysis of the human secretor gene, *FUT2*. The common G428A mutation for secretor (wild-type allele FUT2^*^01; *Se*; dominant) and the non-secretor (mutant allele FUT2^*^01N.02; *se*) of *FUT2* in the European population were tested (Daniels, 2002).

The patient secretor status was determined by molecular analysis of an EDTA blood sample. Genomic DNA (gDNA) was extracted from 350 μL whole blood using a Biorobot EZ1 workstation and commercial EZ1 DNA Blood 350 μL kits (Qiagen, Germany), according to the manufacturer protocol.

The patient secretor status was also determined by qPCR using the Viia7 Real-Time PCR system (Life Technologies, USA), 384-well plates, and TaqMan Genotyping Master Mix (Life Technologies). The qPCR reaction conditions were: 30 s at 60°C (pre-read stage); 10 min at 95°C (hold stage); 40 cycles of 15 s at 95°C; and 1 min at 60°C (PCR stage); and 1 min at 60°C (post-read stage). The qPCR reactions were analyzed using the QuantStudio software, V1.3 (Life Technologies).

The common primers used for *FUT2* were FUT2-F (5′-GGGAGTACGTCCGCTTCAC-3′) and FUT2-R (5′-TGGCGGAGGTGGTGGTA-3′). The specific minor-groove-binder probe for the wild-type allele FUT2^*^01 (*Se*) (428G) was VIC-CTGCTCCTGGACCTT-NFQ, and for the mutant allele FUT2^*^01N.02 (se) (428A) was FAM-CTGCTCCTAGACCTT-NFQ (designed and purchased from Life Technologies). The concentration of the primers in the final qPCR reaction volume was 900 nM, and that of the probes was 250 nM.

The isolated gDNA sample and controls were analyzed in duplicate. The volume of the tested gDNA at concentrations from 50 ng/μL to 100 ng/μL was 2 μL per well. The final volume of the qPCR reactions was 10 μL.

### Estimation of sites under selection

#### P-domain alignment

The alignment of the haplotype deduced amino-acid sequences was performed using the M-Coffee software (http://tcoffee.crg.cat/apps/tcoffee/do:mcoffee; August 2017), with the default settings. The Jalview 2.10 software (Waterhouse et al., 2009) was used for visualization of the alignment (P-domain residues, 289-445) and of the automatically calculated histogram of the conserved physico-chemical properties for each column of alignment (Livingstone and Barton, 1993). The annotations of the common residues of epitopes and the HBGA binding sites of the P domain were based on the details in the literature (see Introduction).

#### Selection pressure on the VP1 gene

Estimations of positive (diversifying) and negative (purifying) selection sites for the aligned haplotypes were performed using Datamonkey (August 2017, Delport et al., 2010). The non-synonymous and synonymous substitution rates were calculated for each codon using five methods: random effects likelihood (REL); mixed effects model of evolution (MEME); fixed effects likelihood (FEL); fast unbiased Bayesian approximation (FUBAR); and single likelihood ancestor counting (SLAC), with the general reversible model (REV) for nucleotide substitution (Kosakovsky Pond and Frost, 2005; Murrell et al., 2012, 2013). The significance level was set to 0.05 for MEME, FEL, and SLAC, and 50 and 0.9 for REL and FUBAR, respectively.

#### Protein modeling

Homodimeric P-domain capsid models were constructed for the haplotypes p1t1_f1.00, p1t7_f0.69, and p1t9_f0.25, using the SWISS-MODEL software (Arnold et al., 2006), based on the template of NV strain Saga4 in complex with HBGA type Le^b^ (tetraglycan) (Singh et al., 2015). The p1t1_f1.00 haplotype model had 98.7% template sequence identity, and all-residue RMSD of 0.098, GMQE of 0.63, and QMEAN of 0.96. The p1t7_f0.69 model had 90.85% template sequence identity, RMSD of 0.211, GMQE 0.61, and QMEAN of 0.42. The p1t9_f0.25 model had 88.56% template sequence identity, RMSD of 0.196, GMQE of 0.60, and QMEAN, 0.53. For visualization and analysis of the structural models, the PyMOL software was used (PyMOL Molecular Graphics System, Version 1.1, LLC).

## Results

### NV typing and quantification

Nine of the 25 stool samples that were taken from July 2009 to June 2014 every 3–10 months were taken through the deep sequencing analysis (Table 1, p1t1-p1t9). The NV RNA concentrations varied from 4.7 × 10^5^ to 1.1 × 10^8^ genome copies/mL 10% (w/v) stool suspension (Table 1), according to the RT-qPCR system developed.

**Table 1:** Samples included in NV molecular analysis with the determined viral genome copy concentration, mean amplicon coverage, and the number of haplotypes constructed.

**Sample code**	**Collection date (yyyy/mm/dd)**	NV concentration (×10^6^ genome copy/mL)^*^	**Mean coverage**	**Constructed haplotypes (n)**
p1t1	2009/07/01	14.3	2,583.5	1
p1t2	2010/04/16	4.8	1,004.5	6
p1t3	2010/09/14	0.7	1,769.3	6
p1t4	2011/06/21	15.8	1,408.0	3
p1t5	2012/01/27	12.9	1,506.9	5
p1t6	2012/11/29	144.5	2,613.2	7^**^
p1t7	2013/06/04	109.6	2,516.3	2
p1t8	2014/03/03	31.6	2,331.8	3^**^
p1t9	2014/06/16	0.5	2,806.7	5

* NV concentration expressed as number × 10^6^ of genome copies per mL of 10% (w/v) stool suspension;

** *one haplotype (from sample p1t6 and p1t8) was constructed with missense codon and thus, was excluded from further analysis—the final total number of constructed haplotype was 36*.

The NV genotype was GII.4_Den Haag 2006 in all of the samples analyzed. During this recorded shedding period of 2,125 days, the 25 samples were analyzed for the presence of enteric pathogens, and all of these were positive for GII NVs. Table 1 gives only the samples that were selected for deep sequencing (p1t1-p1t9), which indicates the high variability of the NV RNA concentrations detected.

### SNP accumulation and VP1 gene haplotypes phylogenetic analysis

**Figure 1:**
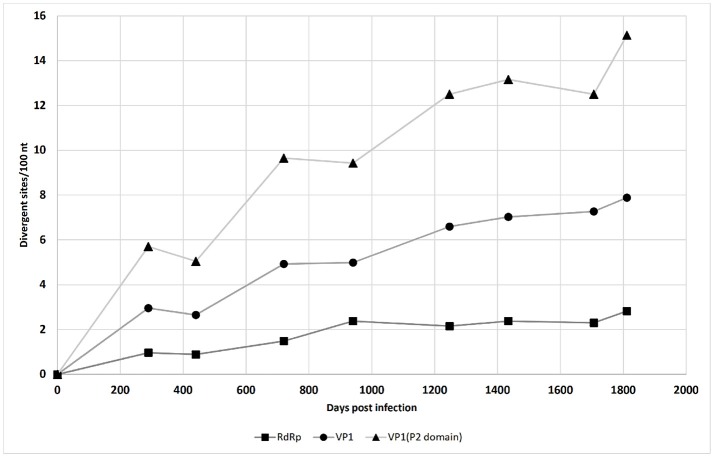
Single nucleotide polymorphism accumulation in the NV RdRp gene region, VP1 gene region, and P2 domain of the VP1 gene region over time (see also Table 1).

**Figure 2:**
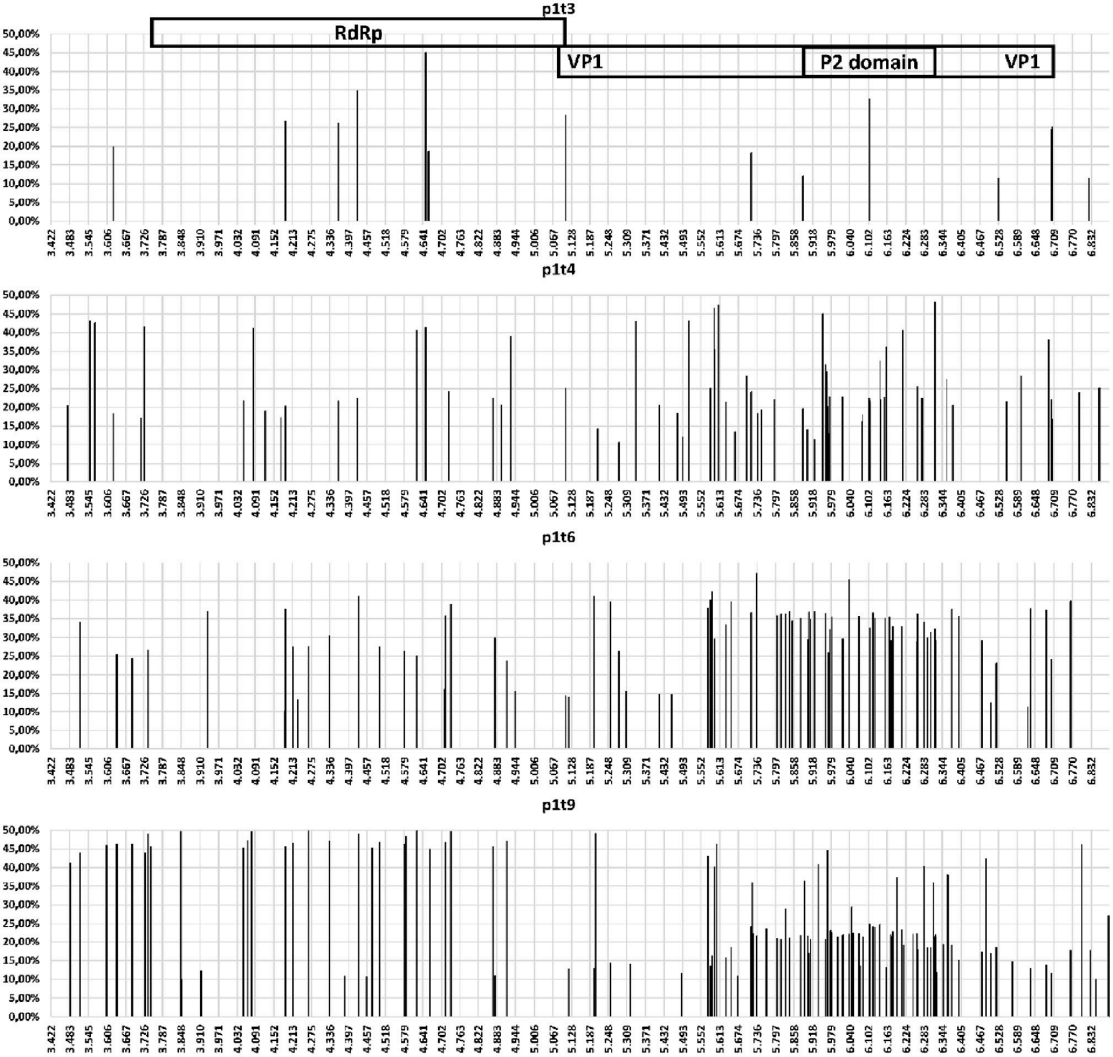
Schematic representation for specific samples (as indicated) for the variable sites (single nucleotide polymorphism occurrence and variant frequency) throughout the amplicon that contained the NV RdRp gene and VP1 gene regions.

The accumulation of mutations was studied first using SNP analysis for comparison of the RdRp and VP1 genes. As shown in Figure 1, the accumulation rate of point mutations was 3-fold greater in the VP1 gene than the RdRp gene. Moreover, the variable sites per 100 nucleotides reached 15.1 for the P2 domain of VP1, followed by 7.9 for the complete VP1, and 2.8 for the RdRp gene. This is shown schematically in the diagram of SNP frequencies in Figure 2. No SNPs were detected in the RdRp and VP1 genome regions in the NV strain sequenced from the first stool sample analyzed, which was obtained from the patient 10 days after the initial onset of diarrhea.

**Figure 3:**
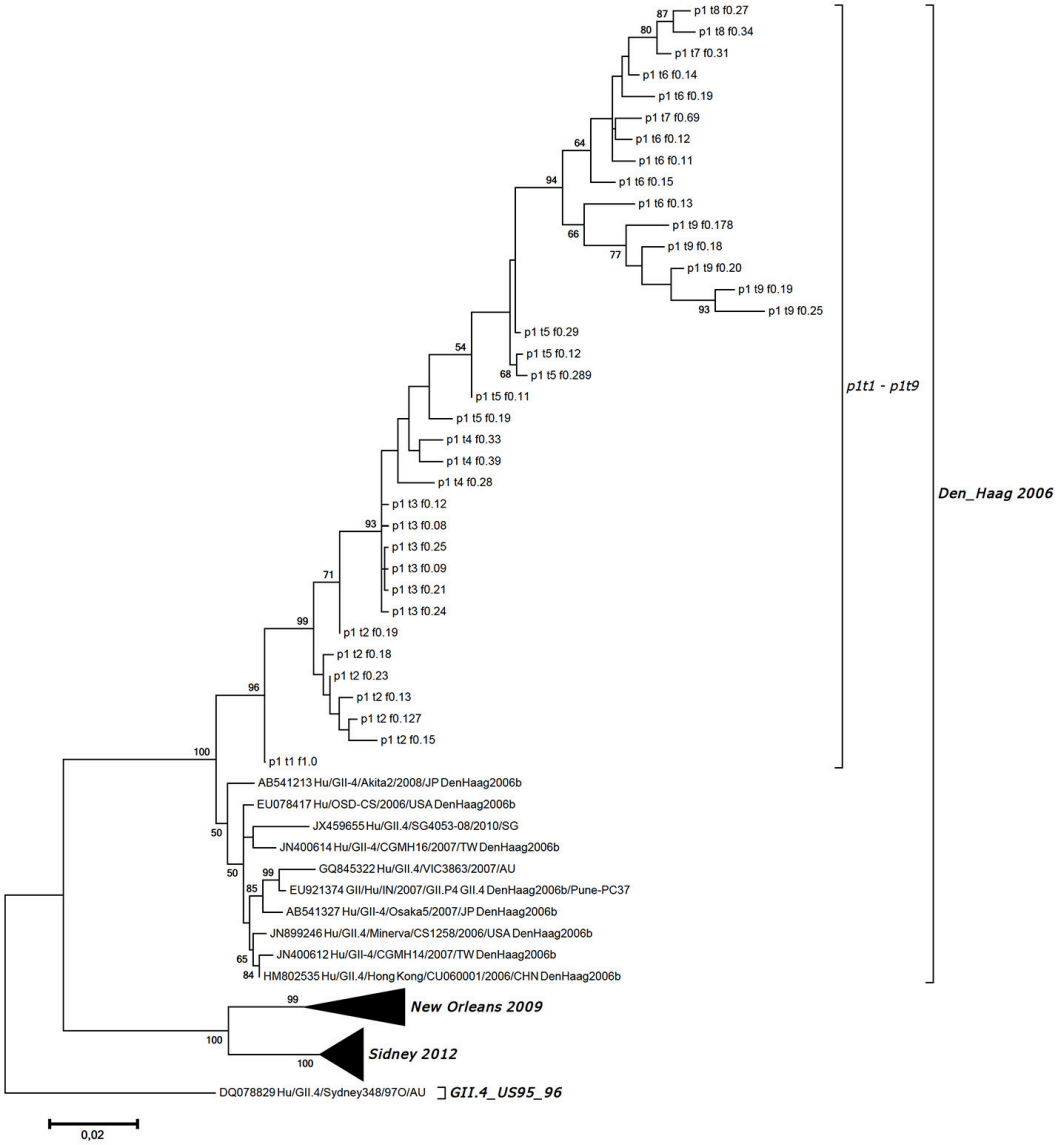
Maximum likelihood phylogenetic tree for the haplotypes generated for the VP1 gene region along with the most identical GII.4 Den_Haag 2006 variant strains from GenBank and other variants for comparison. Haplotype designation is as given in Table 1, as the patient code (p1), the sampling point (t1-t9), and the frequency of the generated sequence (f0.08–f1.00).

In total, we obtained 38 VP1 haplotypes, as presented in Table 1. However, only 36 of them were included in further analysis as two constructed haplotypes contained missense codons. To confirm that there was no subsequent NV infection, all of the 36 haplotype sequences generated were included in the phylogenetic analysis of the NV strains, which also included GenBank-derived strains with high BLAST similarity, and some GII.4 variants other than Den Haag 2006. Figure 3 shows the haplotype phylogenetic segregation of these sequential stool samples. The haplotype branch in the phylogenetic tree was separated with high bootstrap values from the other closely related strains from GenBank, which indicated that there was no additional co-infection with other NV strains (Figure 3). Within each of these sequential samples, the NV haplotypes showed identities from 97.28 to 99.87% for their nucleotide sequences, and from 97.41 to 100.00% for their deduced amino-acid sequences. Between the samples, the identities were much lower: from 93.89 to 99.32% for their nucleotide sequences, and from 92.41 to 99.07% for their deduced amino-acid sequences.

### Patient LE blood group and secretor status

Serological typing for the patient LE blood group showed that she was Le(a^−^b^+^) and consequently had positive secretor status. This was confirmed by molecular analysis. The patient was homozygous (Se/Se) for the secretor locus of the *FUT2* gene.

### Analysis and visualization of selection sites

**Figure 4:**
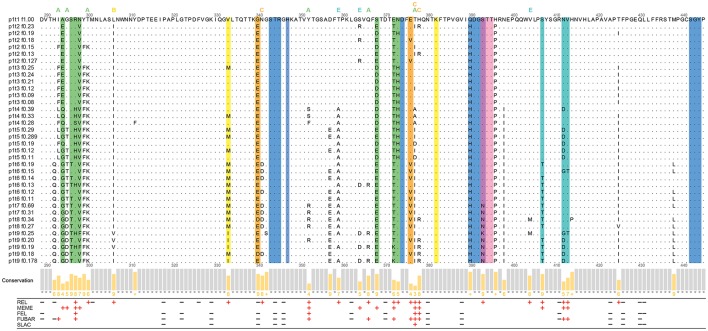
Analysis of the partial P-domain alignment of the haplotypes (residues 289–445). Commonly defined epitopes and HBGA binding sites are highlighted as the colored columns. Green, epitope A; yellow, epitope B; orange, epitope C; pink and violet, epitope D; cyan, epitope E; blue and violet, HBGA binding sites. The sites within the 8-Å expanded area of the epitopes are indicated with letters above the alignment, colored as for the epitopes. The Jalview histogram below the alignment indicates the conservation of the physico-chemical properties for each column (lower bars with lower numbers, lower conservation; completely conserved columns are in gray). The positive and negative selection pressures defined through the Datamonkey algorithms are shown as red plus and black minus signs, respectively.

The deduced VP1 amino-acid sequences of 36 haplotypes (540 amino acids each) were aligned and examined for amino-acid changes. There were a total of 55 non-conserved amino-acid positions in the complete VP1 alignment, and 37 of these non-conserved sites were in the partial P-domain alignment (residues 289-445), as shown in Figure 4. Out of these 37 non-conserved sites in the partial P domain, 15 were within the defined epitopes (Figure 4).

To detect the codons that provide stronger evidence of purifying or diversifying selection, the coding sequences of these 36 haplotypes were examined using Datamonkey. These results are presented at the amino-acid level in Figure 4 (for all 93 negative-selection and 37 positive-selection codons of the aligned VP1 haplotypes, see Supplementary Material Table 1).

For P-domain positions 289-445, negative selection was defined at 19 sites by at least one of the four methods (Figure 4, black minus signs). Positive selection was defined at 25 sites (by at least one of the five methods; Figure 4, red plus signs). Twelve out of these 25 positive selection sites were a part of the known epitopes A, B, C, D, and E (Allen et al., 2009; Debbink et al., 2012, 2014; Lindesmith et al., 2012a,b).

Among the seven positions that were defined as epitope A (Figure 4, green columns), site 294 had the lowest conservation of physico-chemical properties. The site 294, and sites 298, 368, and 373, were defined as under positive selection only by one of the Datamonkey methods, while sites 297 and 372 had the strongest support for positive selection.

Putative epitope B (Figure 4, yellow columns) and putative epitope C (Figure 4, orange columns) were here defined as under positive selection only at site 333 (epitope B) and site 376 (epitope C). Site 376 also had low conservation of physico-chemical properties. On the other hand, site 340 of epitope C was even defined as under negative selection. For epitope D (Figure 4, pink and violet columns), only site 393 was defined as under positive selection.

All three of the residues of epitope E (Figure 4, cyan columns), site 407, and sites 412 and 413, were defined as under positive selection. Site 412 also had the lowest conservation of physico-chemical properties in epitope E.

**Figure 5:**
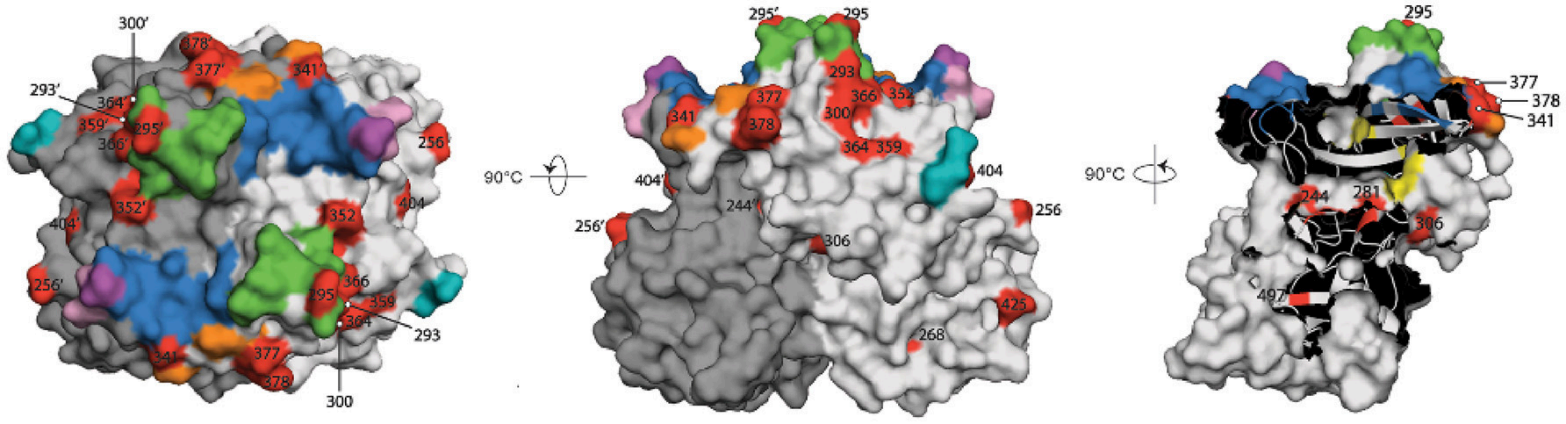
Surface model representations of the P domain. The epitopes and HBGA binding pockets are colored as in Figure 4, and are superimposed over the red sites defined with positive diversifying selection pressure. Black numbers, sites in the chain A monomer (light gray); black numbers with apostrophe, sites in the chain B monomer (dark gray); green, epitope A; yellow, epitope B; orange, epitope C; pink and violet, epitope D; cyan, epitope E; blue and violet, HBGA binding sites. Left, top view; center, side view; right, view of the chain A/B interface.

The remaining 13 of the 25 sites in the partial P-domain alignment (residues 289-445) that were defined as under positive selection by Datamonkey were not a part of the commonly defined epitopes (Figure 4). Along with the other positive selection sites of the P domain (residues 225-530), these sites are highlighted in the three-dimensional surface model representations shown in Figure 5.

Although these 13 sites were not part of the defined epitopes, it has been suggested that other residues within an 8-Å range can influence the structure of epitopes (Lindesmith et al., 2012a; Kulkarni et al., 2016). We therefore analyzed the p1t1_f1.00 haplotype model using PyMOL to determine whether any of these 13 positive selection sites were part of the expanded epitope area (Figure 4, colored letters above the alignment).

In this 8-Å range of expanded epitope A, sites defined as under positive selection were 293, 295, 300, 352, 366, and site 377, which is also part of the expanded area of epitope C (Figures 4, 5). As well as site 377, the expanded range of epitope C included two further sites defined as under positive selection: site 378, and site 341 (Figures 4, 5). The expanded area of epitope E included three further sites defined as under positive selection: sites 359, 364, and 404 (Figures 4, 5).

Epitope B is positioned at the interface between the two monomers, and its expanded area included sites 281 (not in the range of alignment in Figure 4), 306, and 244′ (on the second monomer), which were defined as under positive selection (Figure 5).

Finally, there were positive selection sites in the P domain that were not part of any of these commonly defined epitopes, nor part of the expanded epitope areas. These included four sites defined as under positive selection: site 256, and sites 268, 425, and 497 (a buried residue) (Figure 5). There were no positive selection residues in the expanded range of epitope D.

### Modeling of HBGA binding

The analysis of the HBGA binding pocket residues (Figure 4, blue columns) defined site 344 as under negative selection, and site 393 (Figure 4, violet column) as under positive selection. We examined the potential influence of changes at site 393 on Le^b^ HBGA binding [with the patient phenotype of Le(a^−^b^+^)].

**Figure 6:**
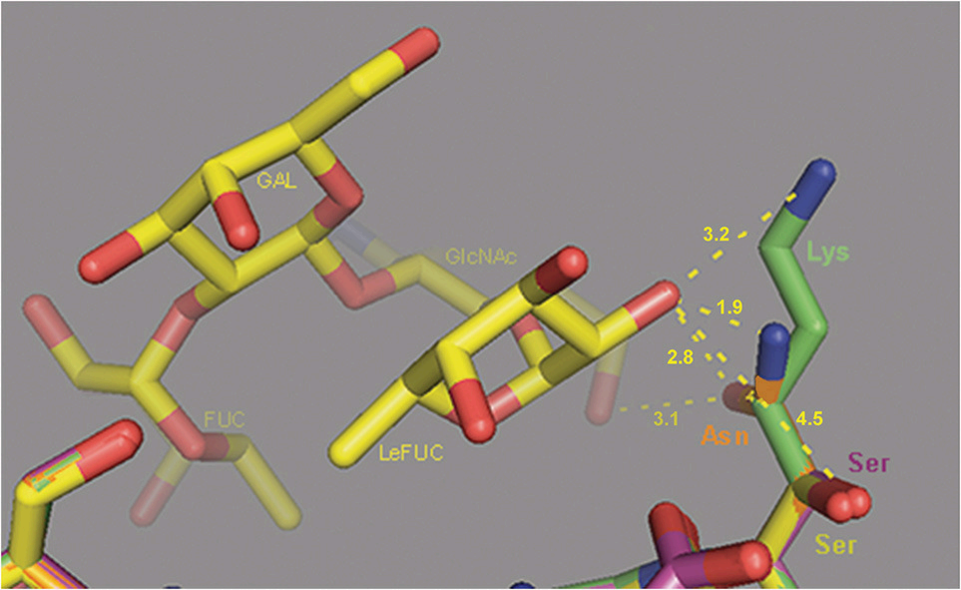
Molecular modeling of the interactions of VP1 site 393 with Le^b^ HBGA. Superposition of the 4OPO crystal structure of strain Saga4 in complex with HBGA type Le^b^ tetraglycan (yellow sticks), and the models of p1t1_f1.00 (violet sticks), p1t7_f0.69 (orange sticks), and p1t9_f0.25 (green sticks). Ser, serine; Asn, asparagine; Lys, lysine; FUC, fucose; LeFUC, Lewis fucose; GlcNAc, N-acetylglucosamine; GAL, galactose. Dashed lines show distances from side chain atoms of residues at site 393 to LeFUC and GlcNAc.

In addition to the p1t1_f1.00 haplotype model, we also constructed the models p1t7_f0.69 and p1t9_f0.25, which were based on the 4OPO crystal structure of strain Saga4 in complex with HBGA type Le^b^ tetraglycan (Singh et al., 2015). The superpositions of the 4OPO structure (Figure 6, yellow lines), and the models p1t1_f1.00 (Figure 6, violet lines), p1t7_f0.69 (Figure 6, blue lines), and p1t9_f0.25 (Figure 6, green lines), showed that the Ser393 side chain in 4OPO template and in p1t1_f1.00 model was pointing away from Le^b^ tetraglycan. Also, at 4.45 Å in distance, it cannot form hydrogen bonds with the C2 oxygen of Lewis fucose (LeFUC; Figure 6, LeFUC). In contrast, the Asn393 of the p1t7_f0.69 model was positioned closer to LeFUC and N-acetylglucosamine (GlcNAc; Figure 6, LeFUC, GlcNAc) and can form putative hydrogen bonds with the oxygens (2.8 Å to C2 oxygen of LeFUC; 3.1 Å to C6 oxygen of GlcNAc), or with the NH_2_ group of its side-chain amide (1.9 Å to C2 oxygen of LeFUC). In the p1t9_f0.25 model, the Lys393 side-chain pointed upward from the VP1 backbone and the Le^b^ tetraglycan, but its ε-amino group was 3.2 Å from the C2 oxygen of LeFUC (Figure 6, LeFUC), and can therefore form a putative hydrogen bond. The positions and interactions of residues 344, 345, 374, 391, 392, 442, and 443, which are also involved in Le^b^ tetraglycan binding (Singh et al., 2015), were conserved between 4OPO, p1t1_f1.00, p1t7_f0.69, and p1t9_f0.25 (data not shown).

## Discussion

NVs are an important cause of diarrhea in transplant recipients and are responsible for prolonged or chronic diarrhea in patients with immunodeficiency (Schorn et al., 2010; Angarone et al., 2016; Echenique et al., 2016; Lee et al., 2016). In the literature, NV infections in immunocompromised patients after transplantation has been described as a chronic symptomatic infection with prolonged moderate diarrhea, or as an acute symptomatic phase that can be followed by extended asymptomatic shedding of NVs (Echenique et al., 2016). It has also been reported that in chronic infections, NV replication takes place in enterocytes (Karandikar et al., 2016). This indicates that the NVs are produced and shed in stools of chronic infected patients, which might consequently present a source of infection. Indeed, NVs are excreted in relatively high concentrations in stool samples of chronically infected persons (Ludwig et al., 2008).

This was shown also for the present patient, as the concentration of NV genome copies was in the range of 10^6^–10^8^/mL in 10% (w/v) stool suspension. Interestingly, the patient developed symptoms of diarrhea only for the initial days of the NV infection, which then disappeared without remission. This might reflect moderate immune protection for this patient, although without the potential for NV clearance. This situation might also have contributed to selective pressure that would then generate genetic, and consequently antigenic, variants, which has been reported previously in other studies (Siebenga et al., 2008; Debbink et al., 2014), and is confirmed by the present data.

Here, the mutation rate was not equally distributed throughout the NV genome, but was greater in the gene coding for the NV capsid protein VP1. The P domain is part of the NV capsid that includes antibody blockade epitopes, and its sites are therefore under strong diversifying selection pressure (Kobayashi et al., 2016), which can generate new epidemic NV variants (Bull et al., 2012; Lindesmith et al., 2013). The present study also showed concentration of SNPs in the P2 region, which had the highest rate of amino-acid changes.

The phylogenetic analysis of the NV haplotypes constructed for the VP1 nucleotide sequences indicated that the NV variants detected might not be strains from subsequent infections, but appeared to be as a result of the accumulation of mutations of the initial NV haplotype within the host. The generation of NV genetic and antigenic variants with more frequent and intense mutation rates in the genome region of the capsid protein gene is probably the consequence of NV–host interplay and selective pressure, rather than being randomly observed mutations. As a limitation to our study, it should be considered that subsequent exposure and infection with genetically close NV variants could not be absolutely ruled out. The patient was not isolated, and may have been consequently exposed to circulating NV strains.

Sixty-seven percent of the non-conserved sites that were defined as under positive selection in the full-length VP1 protein alignment were between residues 289 and 445 of the P domain. About half of these residues in this partial P-domain alignment did not belong to the commonly defined epitopes. There were six residues under positive selection (residues 293, 295, 300, 352, 366, 377) that might influence the epitope A structure as they were in the 8-Å expanded area (Lindesmith et al., 2012a). All of these sites have already been described as variable and indicated as candidates of the escape phenotype (Bull et al., 2012; Lindesmith et al., 2012a; Debbink et al., 2014; Kulkarni et al., 2016). In particular, sites 352 and 377 had strong support as putative antibody-recognition residues in the present study. On the other hand, site 296 has been generally defined as part of epitope A (Debbink et al., 2014), but it was not defined as under positive selection. Bull et al. (2012) indicated that site 296 was completely conserved in their report on intrahost NV evolution.

It has been proposed that the changes in the epitope B residues that have been positioned at the interface of the monomers might have a role in the evolution of novel strains by compensating for, and therefore allowing, larger physico-chemical variations of surface-exposed residues (Donaldson et al., 2008; Lindesmith et al., 2012a). Our results supported this theory, as site 333 and the epitope B expanded area sites under positive selection were all at the interface of the two monomer chains. Site 340 of epitope C, here defined as under negative selection, is a part of a turn in the polypeptide backbone, which protrudes from the surface proximal to the HBGA binding pocket. This negative selection pressure on site 340 might indicate its role in stabilization of the protein structure. In contrast, other sites of epitope C, including the expanded area sites, were under strong positive selection; these have also been described as variable sites in other studies (Bull et al., 2012; Kulkarni et al., 2016).

Only site 393 of epitope D was defined as under positive selection, and this site (Ser393) has also been reported to be involved in binding of the fucose moiety of Le^b^ HBGA in the GII.4 strain TCH05 (Shanker et al., 2011). However, the recently solved crystal structure of strain Saga4 in complex with HBGA type Le^b^ (to 1.4 Å resolution) revealed a different orientation of Ser393, which did not form hydrogen bonds with LeFUC (Singh et al., 2015). Ser393 was also present in all of the haplotypes in our study until sampling point p1t7, where it was replaced by Arg, and in haplotype p1t9 f0.25, where it was replaced by Lys. The side-chains of Arg and Lys were positioned closer to Le^b^ tetraglycan in the models, and might therefore make hydrogen bonds. To speculate, the flexible side-chain of Lys might even compensate for multiple HBGA binding positions. All of the other sites of epitope D were conserved in the present alignment. Also, the expanded area of epitope D did not include any residues under positive selection. This conservation can be explained at least in part by the vicinity of the HBGA binding pocket: as the 8-Å expanded area of epitope D covers the majority of the HBGA residues (390-392, 443-446), any variations in these epitope residues might influence the structure of the HBGA binding pocket (Lindesmith et al., 2012a). However, although there was high conservation in this region, none of the residues of epitope D and its expanded area were defined as under negative selection.

All of the common residues of epitope E (Lindesmith et al., 2012b) were defined as under positive selection in the present alignment of the evolving haplotypes. For the expanded area of epitope E, all residues under positive selection defined here were already indicated as putative epitope residues in other studies (Debbink et al., 2014; Kobayashi et al., 2016; Kulkarni et al., 2016).

To conclude, this report confirms the importance of NV infection in immunocompromised patients, both in terms of a constant source of possible infection and an opportunity to study the molecular evolution of NVs. The present study has defined new putative candidates for the escape phenotype in the evolving NV in the host. According to our bioinformatic analysis, we identified residues that had not been defined previously as parts of the known epitopes, but were under strong positive selection. The positions of the most important residues form an arch that extends from epitope C (residues 377, 378), goes below epitope A (sites 300, 366), and ends proximal to the HBGA pocket (site 352). Our observations of intrahost NV evolutionary dynamics and the consequent amino-acid changes most likely indicates interplay between immune-driven mutations and optimization of NV binding to HBGA, as proposed previously by Shanker et al. (2011). As both fucosyl moieties of Le^b^ have been implicated in hydrogen bonding (Shanker et al., 2011), which has been shown in particular for GII.4 genotype strains, the Lewis and secretor status might be important for the outcome of NV infections in immunocompromised patients. It would be interesting to see whether Le-positive and secretor-positive individuals [i.e., Le(a^−^b^+^)] with both fucosyl moieties are at higher risk of developing chronic infection while under immunosuppression than those patients with only one Le-positive, secretor-negative [i.e., Le(a^+^b^−^), or Le-negative, secretor-positive (not secretor-negative; i.e., Le(a^−^b^−^)]. Immediate intervention or possible antivirals, like nitazoxanide, might help prevent chronic NV infection and shedding, although this needs further investigation.

## Author contributions

AS, TK, AŠ, MA, TD-D, and MP-P designed the study, AS, MS, MK, JG, and MP-P were responsible for molecular analysis of the detected NV strains, NGS analysis and analysis of NGS data, development of molecular detection and quantification method. TK performed protein modeling and virus-HBGA binding analysis. TD-D performed HBGA typing with antigen test, developed and performed genetic test for secretor status and molecular HBGA typing. AŠ and MA collected and analyzed clinical data and were responsible for clinical part of the study. AS, TK, AŠ, JG, and TD-D collected the results and wrote the paper. All authors read and critically reviewed the manuscript.

### Conflict of interest statement

The authors declare that the research was conducted in the absence of any commercial or financial relationships that could be construed as a potential conflict of interest.
