# Supplementary material for: Intrahost Norovirus Evolution in Chronic Infection Over 5 Years of Shedding in a Kidney Transplant Recipient
Source: Front Microbiol. 2018 Mar 2;9:371. doi: 10.3389/fmicb.2018.00371 (PMC5840165; doi:10.3389/fmicb.2018.00371)
Supplement: Supplementary file 1 [file Table1.docx]

**Supplementary Materials Table 1:** Details of the modeling of all of the 93 negative and 37 positive selection codons (sites) of the aligned VP1 haplotypes, including the positive selection sites reported by Kobayashi et al. (2016). REL, random effects likelihood model; MEME, mixed effects model of evolution; FEL, fixed effects likelihood model; FUBAR, fast unbiased Bayesian approximation model; SLAC, single likelihood ancestor counting model.

| **Codon/** | **Algorithm** | | | | | **Kobayashi et al.** |
| --- | --- | --- | --- | --- | --- | --- |
| **site** | **REL** | **MEME** | **FEL** | **FUBAR** | **SLAC** | **(2016)** |
| 1 | **–** |  |  |  |  |  |
| 3 | **–** |  |  |  |  |  |
| 6 |  |  |  |  |  | **+** |
| 8 | **–** |  | **–** | **–** | **–** |  |
| 9 |  |  |  |  |  | **+** |
| 12 | **+** |  |  |  |  |  |
| 16 |  |  |  |  |  | **+** |
| 23 |  |  |  |  |  | **+** |
| 25 |  |  |  |  |  | **+** |
| 27 | **–** |  |  |  |  |  |
| 33 |  | **+** |  |  |  |  |
| 37 | **–** |  |  |  |  |  |
| 39 | **–** |  | **–** | **–** | **–** |  |
| 41 | **–** |  | **–** | **–** | **–** |  |
| 57 | **–** |  | **–** | **–** | **–** |  |
| 64 |  |  |  |  |  | **+** |
| 66 | **–** |  | **–** | **–** |  |  |
| 74 | **–** |  |  |  |  |  |
| 75 | **–** |  |  |  |  |  |
| 79 | **–** |  | **–** | **–** |  |  |
| 85 | **–** |  | **–** | **–** |  |  |
| 95 | **–** |  |  |  |  |  |
| 104 | **–** |  | **–** | **–** | **–** |  |
| 107 | **–** |  | **–** | **–** |  |  |
| 110 | **–** |  | **–** | **–** | **–** |  |
| 120 | **–** |  |  |  |  |  |
| 124 | **–** |  | **–** | **–** | **–** |  |
| 130 | **–** |  |  |  |  |  |
| 134 | **–** |  |  |  |  |  |
| 140 | **–** |  |  |  |  |  |
| 141 | **–** |  |  |  |  |  |
| 143 | **–** |  | **–** | **–** | **–** |  |
| 144 | **–** |  |  |  |  |  |
| 145 | **–** |  |  |  |  |  |
| 149 | **–** |  |  |  |  |  |
| 164 | **–** |  | **–** | **–** | **–** |  |
| 166 | **–** |  | **–** | **–** | **–** |  |
| 168 | **–** |  | **–** | **–** | **–** |  |
| 171 | **+** | **+** | **+** | **+** |  |  |
| 173 | **–** |  | **–** | **–** |  |  |
| 175 | **–** |  | **–** | **–** |  |  |
| 179 | **–** |  |  |  |  |  |
| 181 | **–** |  |  |  |  |  |
| 182 | **–** |  |  | **–** |  |  |
| 183 | **–** |  | **–** | **–** | **–** |  |
| 189 | **–** |  | **–** | **–** | **–** |  |
| 193 |  |  |  | **+** |  |  |
| 206 | **–** |  | **–** | **–** |  |  |
| 211 | **–** |  | **–** | **–** | **–** |  |
| 212 | **–** |  |  |  |  |  |
| 213 | **–** |  | **–** | **–** | **–** |  |
| 217 | **–** |  | **–** | **–** |  |  |
| 221 | **–** |  |  |  |  |  |
| 222 | **–** |  | **–** | **–** | **–** |  |
| 236 | **–** |  | **–** | **–** |  |  |
| 237 | **–** |  |  |  |  |  |
| 239 | **–** |  | **–** | **–** | **–** |  |
| 244 | **+** |  |  | **+** |  |  |
| 245 | **–** |  | **–** | **–** | **–-** |  |
| 248 | **–** |  | **–** | **–** | **–** |  |
| 253 | **–** |  | **–** | **–** | **–** |  |
| 256 | **+** | **+** |  |  |  |  |
| 257 | **–** |  | **–** | **–** | **–** |  |
| 265 | **–** |  | **–** | **–** |  |  |
| 268 | **+** |  |  |  |  | **+** |
| 269 | **–** |  | **–** | **–** | **–** |  |
| 272 | **–** |  | **–** | **–** | **–** |  |
| 273 | **–** |  |  | **–** |  |  |
| 274 | **–** |  | **–** | **–** | **–** |  |
| 276 | **–** |  | **–** | **–** |  |  |
| 281 |  |  |  | **+** |  |  |
| 289 | **–** |  | **–** | **–** |  |  |
| 292 | **–** |  |  | **–** |  |  |
| 293 |  |  |  | **+** |  |  |
| 294 |  | **+** |  |  |  |  |
| 295 |  | **+** |  |  |  |  |
| 297 | **+** | **+** | **+** | **+** |  | **+** |
| 298 |  | **+** |  |  |  | **+** |
| 300 | **+** |  |  |  |  |  |
| 301 | **–** |  |  |  |  |  |
| 303 |  |  |  |  |  | **+** |
| 306 | **+** |  |  |  |  |  |
| 318 | **–** |  | **–** | **–** | **–** |  |
| 322 | **–** |  | **–** | **–** |  |  |
| 326 | **–** |  |  |  |  |  |
| 329 | **–** |  | **–** | **–** | **–** |  |
| 333 | **+** |  |  |  |  |  |
| 340 | **–** |  | **–** | **–** | **–** |  |
| 341 | **+** |  |  |  |  |  |
| 344 | **–** |  | **–** | **–** | **–** |  |
| 346 | **–** |  | **–** | **–** | **–** |  |
| 352 | **+** | **+** | **+** | **+** |  |  |
| 359 | **+** |  |  |  |  | **+** |
| 360 |  |  |  |  |  | **+** |
| 362 | **–** |  | **–** | **–** |  |  |
| 364 |  | **+** |  |  |  |  |
| 366 | **+** |  |  | **+** |  |  |
| 368 | **+** | **+** |  | **+** |  |  |
| 370 | **–** |  | **–** | **–** |  | **+** |
| 372 | **+** | **+** |  | **+** |  |  |
| 373 | **+** |  |  |  |  |  |
| 376 | **+** |  |  | **+** |  |  |
| 377 | **+** | **+** | **+** | **+** | **+** |  |
| 378 | **+** | **+** | **+** | **+** |  |  |
| 379 |  |  |  |  |  | **+** |
| 380 | **–** |  | **–** | **–** | **–** |  |
| 388 | **–** |  | **–** | **–** | **–** |  |
| 393 | **+** |  |  |  |  |  |
| 397 |  |  |  |  |  | **+** |
| 404 | **+** |  |  |  |  |  |
| 407 | **+** | **+** |  |  |  |  |
| 411 | **–** |  | **–** | **–** |  |  |
| 412 | **+** | **+** | **+** | **+** |  |  |
| 413 | **+** | **+** |  | **+** |  |  |
| 416 |  |  |  |  |  | **+** |
| 419 |  |  |  |  |  | **+** |
| 423 | **–** |  | **–** | **–** | **–** |  |
| 425 | **+** |  |  |  |  |  |
| 426 | **–** |  | **–** | **–** | **–** |  |
| 430 | **–** |  | **–** | **–** |  |  |
| 431 | **–** |  | **–** | **–** | **–** |  |
| 435 |  |  |  |  |  | **+** |
| 447 | **–** |  |  |  |  |  |
| 463 | **–** |  | **–** | **–** | **–** |  |
| 473 | **–** |  | **–** | **–** |  |  |
| 479 | **–** |  | **–** | **–** |  |  |
| 484 | **–** |  | **–** | **–** | **–** |  |
| 485 |  |  |  |  |  | **+** |
| 487 | **–** |  |  |  |  |  |
| 490 | **–** |  |  | **–** |  |  |
| 497 | **+** |  |  |  |  |  |
| 503 | **–** |  | **–** | **–** | **–** |  |
| 506 | **–** |  | **–** | **–** | **–** |  |
| 508 | **–** |  | **–** | **–** |  |  |
| 509 | **–** |  | **–** | **–** | **–** |  |
| 513 | **–** |  |  | **–** |  |  |
| 514 | **–** |  | **–** | **–** |  |  |
| 516 | **–** |  | **–** | **–** | **–** |  |
| 517 | **–** |  |  |  |  |  |
| 530 | **–** |  |  |  |  |  |
| 534 | **+** |  |  |  |  |  |
| 536 | **–** |  | **–** | **–** | **–** |  |
| 537 | **+** |  |  |  |  |  |
| 539 | **+** | **+** | **+** | **+** |  |  |
